# Lack of gamma delta T cells ameliorates inflammatory response after acute intestinal ischemia reperfusion in mice

**DOI:** 10.1038/s41598-021-96525-y

**Published:** 2021-09-20

**Authors:** Dominik Funken, Yi Yu, Xiaoyan Feng, Tawan Imvised, Faikah Gueler, Immo Prinz, Omid Madadi-Sanjani, Benno M. Ure, Jochen F. Kuebler, Christian Klemann

**Affiliations:** 1 Department of Pediatric Pneumology, Allergy and Neonatology, Hannover Medical School, Carl-Neuberg-Straße 1, 30625 Hannover, Germany; 2 Department of Pediatric Surgery, Center of Surgery, Hannover Medical School, Hannover, Germany; 3 Department of Nephrology, Hanover Medical School, Hannover, Germany; 4 Institute of Immunology, Hannover Medical School, Hannover, Germany; 5 Institute of Systems Immunology, University Medical Center Hamburg-Eppendorf, Hamburg, Germany; 6 Present Address: Department of Pediatric Surgery, University of Leipzig Medical Center, Leipzig, Germany

**Keywords:** Experimental models of disease, Paediatric research

## Abstract

T-cells have been demonstrated to modulate ischemia–reperfusion injury (IRI) in the kidney, lung, liver, and intestine. Whereas most T-cell subpopulations contribute primarily to the antigen-specific effector and memory phases of immunity, γδ-T-cells combine adaptive features with rapid, innate-like responses that can place them in the initiation phase of immune reactions. Therefore, we aimed to clarify the role of γδ-T-cells in intestinal IRI. Adult *wild-type* (WT) and γδ-*T-cell-deficient* mice were subjected to acute intestinal IRI. Gene expression of pro-inflammatory cytokines and influx of leukocyte subpopulations in the gut were assessed by qPCR and flow cytometry. Serum transaminases were measured as an indicator of distant organ IRI. Intestinal IRI led to increased influx of neutrophils, pro-inflammatory cytokine expression and LDH/ALT/AST elevation. Selective deficiency of γδ-T-cells significantly decreased pro-inflammatory cytokine levels and neutrophil infiltration in the gut following IRI compared to controls. Furthermore, γδ-T-cell deficiency resulted in decreased LDH and transaminases levels in sera, indicating amelioration of distant organ injury. Increasing evidence demonstrates a key role of T-cell subpopulations in IRI. We demonstrate that γδ-T-cell deficiency ameliorated pro-inflammatory cytokine production, neutrophil recruitment and distant organ injury. Thus, γδ-T-cells may be considered as mediators contributing to the inflammatory response in the acute phase of intestinal IRI.

## Introduction

Ischemia/reperfusion injury causes significant morbidity and mortality and occurs in a plethora of clinical conditions such as organ transplantation, resection, trauma, and septic- or hemorrhagic shock. In order to minimize the negative effects of IRI, the complex pathogenesis has to be elucidated. Inflammation has been identified as one key factor. Initially, tissue facors and the innate immune system were thought to mediate the inflammatory response following IRI. However, emerging data demonstrated a role of T cells as mediators of IRI^1–6^. A number of studies firmly established conventional αβ T cells as contributors to IRI in organs such as the liver and kidney. In intestinal IRI, however, the role of T cells remains controversial. Studies with SCID mice, which lack all T- and B-cells, have implicated an amelioration of intestinal IRI by a lack of T cells^7,8^. However, we have recently demonstrated that selective deficiency of conventional αβ T cells did not impact the damage in intestinal IRI^9^. Therefore, we hypothesized that the lack of γδ T cells might be responsible for the observed amelioration of IRI in SCID mice. γδ T cells are a unique T cell subset and among the very first T cells to develop in all vertebrates^10–13^. In the circulation, γδ T cells represent only a minor fraction of T cells, but tissue-resident epithelial γδ T cells represent the major T cell population in the epithelium of the gut^14^. γδ T cells combine features of the adaptive immune system, such as undergoing thymic selection and expression of a T cell receptor, with features of the innate immune system such as the capacity for pattern- or danger signal recognition in order to initiate rapid immune responses^15,16^. Of note, in IRI models of kidney and brain γδ T cells have been shown to aggravate ischemic injury by generating chemotactic signals for peripheral myeloid cells such as neutrophils and monocytes^17,18^. Thus, in the study at hand, we aimed to clarify the role of γδ T cells in intestinal IRI. Utilizing a mutant mice strain which selectively lacks γδ T cells, we demonstrate that deficiency of this conserved lymphocyte population results in an amelioration of the inflammatory response in the first hours of acute intestinal IRI.

## Materials and methods

### Animals and model of intestinal IRI

All procedures were approved by the local animal welfare committee at the University of Veterinary Medicine Hannover (permit number 42502-04-12/0769). All methods in the study were carried out in accordance with the Helsinki guidelines and declaration or any other relevant guidelines. Furthermore, the study was carried out in compliance with the ARRIVE guidelines.

For all experiments, 4 weeks old male *C57BL/6 J wild-type* (WT) and mice deficient of γδ T cells (B6.129P2)-T*crd*^*tm1Mom*^) (mean body-weight 15 g) were used^19^. In brief, in B6.129P2-T*crd*^*tm1Mom*^ mice the gamma-delta cell receptor expression in all adult lymphoid and epithelial organs is deficient, while the alpha–beta T-cell lineage show unaffected development.

In order to reduce a possible influence of gut microbiota, all animals were housed in the same room of the facility and littermates were used. Intestinal IRI was induced as previously described^20^. In brief, mice were anesthetized with a combination of Ketamine (100 mg/ml, Albrecht, Germany) and Xylazine (2%, Bayer HealthCare, Germany) by i.p*.* injection. Normothermia was maintained, oxygen supplemented, and for prevention of dehydration, 4 ml/kg/h isotonic saline was injected subcutaneously. Ischemia was induced by clamping the superior mesenteric artery for 30 min with spring-loaded bulldog microvascular clamps (Aesculap, Germany) after midline laparotomy. The celiac trunk was preserved during the whole procedure, to reduce ischemic injury of the liver. Observation of pulsatile mesenteric flow was confirmed before closing the abdominal cavity confirmed reperfusion. After 4 h of surgery, animals were sacrificed, and small intestinal tissue and serum were collected. Previous studies have presented a highly variable degree of injury, following IRI in rodents, in the histology. Different segments of the intestine seem to be more or less sensitive for ischemic injury^21^. Therefore, histological quantification of the intestinal injury was not performed. Aged matched animals undergoing the same procedure except for clamping of the mesenteric artery served as controls in every experiment.

### mRNA extraction and assessment of cytokine expression by quantitative reverse transcription-polymerase chain reaction (qPCR)

mRNA extraction and assessment of cytokine expression by quantitative PCR was performed as previously described^20,21^*.* Briefly, total RNA was extracted from lysed gut tissue using RNeasy Mini Kit (Qiagen, Venlo, The Netherlands). A high-capacity RNA-to-complementary DNA Kit (Applied Biosystems, Foster City, CA) was used for complementary DNA preparation. All transcripts were assessed by QuantiTect Primer Assay (Qiagen) and Maxima SYBR Green/Rox qPCR MasterMix (Thermo Scientific, Waltham, MA) on an Applied Biosystems StepOnePlus Real-Time PCR System (Life Technologies, Carlsbad, CA). Relative gene expression values were normalized to Actin, glyceraldehyde-3-phosphate dehydrogenase, and Hypoxanthine–guanine phosphoribosyltransferase as housekeeping genes.

### Isolation of intestinal leukocytes and flow cytometry

Intraepithelial leukocytes (IEL) and lamina propria leukocytes (LPL) were isolated and processed as previously described^20,21^. Briefly, intraepithelial leukocytes were isolated by incubating cleansed and mechanically homogenized and incubated in PBS with 10% fetal calf serum (FCS) (PAA Laboratories, Cölbe, Germany) in supplemented with 5 mM EDTA (Applichem, Darmstadt, Germany) on an orbital shaker. Subsequently, lamina propria cells were isolated after digestion with 100 µg/mL Liberase (Roche, Basel, Switzerland) and enriched by discontinuous 40%/70% Percoll (GE Healthcare, Buckinghamshire, United Kingdom) density gradient centrifugation. Estimation of absolute cell numbers and flow cytometric analysis were performed according to standard protocols as described previously^21,22^*.* Briefly, fixable Viability Dye (eBioscience, Santa Clara, CA) was used to exclude dead cells. Unspecific binding was prevented by incubation with anti-FcgRII/III mononuclear antibody (Clone: 2.4G2; Biolegend, San Diego, CA). Specific stainings were performed with the following antibody clones and conjugated fluorochromes (all eBiosciences), for T cells: CD45-30-F11-eFluor450, CD3e-145-2C11-PerCP-Cy5.5, CD4-RM4-5-APC-eFluor780, CD8a-53-6.7-FITC, TCRβ-H57-597-APC, TCRγδ-eBioGL3-PE, and for myeloid cells CD45-30-F11-eFluor450 CD11b-M1/70-APC-efluor780, CD11c-N418-PerCP-Cy5.5, Ly6-G-1A8-PE-Cy7, Ly-6C-HK1,4-PE. Samples were acquired on a FACS Canto II flow cytometer with BD FACSDiva software (BD Biosciences, Becton, Dickinson and Company, USA, Version 8.0.1, https://www.bdu.edu/flow-cytometry/files/2010/10/BDFACSDivaSoftwareReferenceManual.pdf) and analyzed with KALUZA software (Beckman Coulter, Brea, USA, Version: 1.5.20365.16139, https://www.beckman.de/resources/videos/products/kaluza-software-performance). Myeloid subpopulations were assessed as described by Rose et al. ^23^*.* and T cells as previously described by our group^21^.

### Assessment of liver enzymes in serum

Lactatdehydrogenase (LDH), Alanine transaminase (ALT) and aspartate transaminase (AST) were measured by using an AU 400 Olympus Analyzer (Olympus, Tokyo, Japan) as previously described^24^.

### Statistics

One-way analysis of variance with a Tukey’s multiple comparisons test was performed with GraphPad Prism software, v6.0 (GraphPad Software, San Diego, CA, USA). Data are displayed as mean and SD with p < 0.05 considered as statistically significant.

## Results

### Ameliorated leukocyte influx following IRI in γδ T cell deficient mice

**Figure 1:**
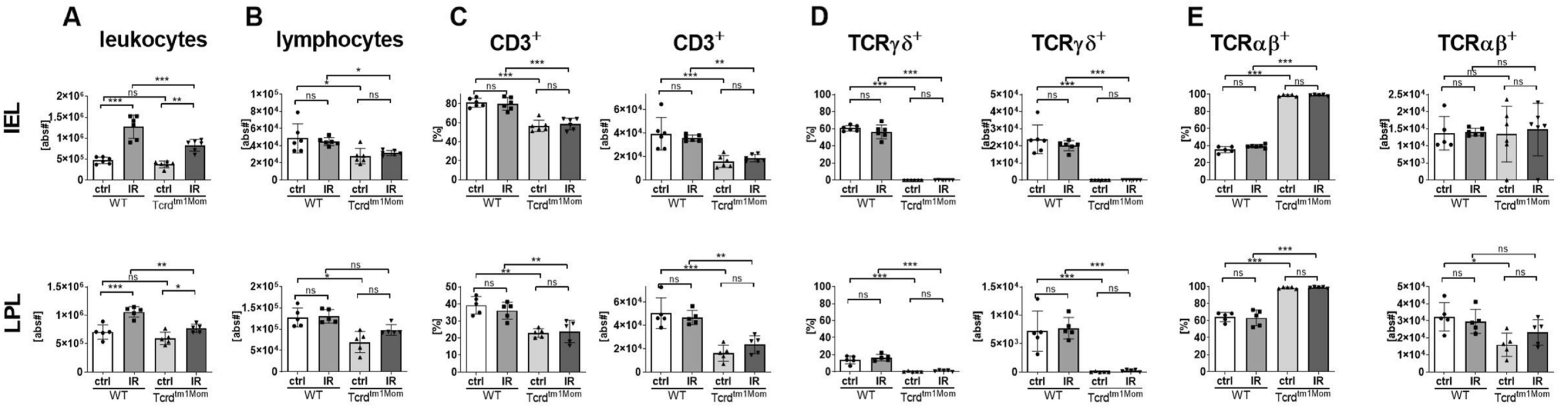
Attenuated leukocyte influx into the gut following IRI in γδ T cell deficient mice.** (A)** Leukocyte absolute numbers (live CD45^+^ cells) in the epithelium of the small intestine (IEL, upper row) and the lamina propria (LPL, lower row) of wild-type (WT) mice and mice deficient of γδ T cells (Tcrd^tm1Mom^) after intestinal ischemia reperfusion (IR) or sham operation (ctrl). **(B)** Absolute lymphocyte numbers were assessed by forward and sideward scatter characteristics of live CD45^+^ cells in flow cytometric analyses and calculated from total leukocyte numbers. A minimum of 1 × 10^5^ living leukocytes per sample were analyzed by flow cytometry. **(C)** Percentage of total lymphocytes displayed in **(B)** positive for the pan T cell marker CD3. **(D)** Percentages of the gamma delta T cells among CD3 + T cells. **(E)** Percentages of CD3^+^ T cells expressing the alpha beta T cell receptor. Each data point represents the results from an individual mouse. Bars represent group mean and error bar depicts ± SD. N = 5–6/group. *p < 0.05; **p < 0.01; ***p < 0.001. *ns* not significant. Data are representative of two independent experiments.

In order to elucidate the role of gamma delta T cells in acute intestinal IRI, we performed flow cytometric T cell phenotyping of lymphocytes isolated from the gut of WT mice and mice lacing γδ T cells. Assessment of total cell numbers showed an increase of leukocytes following IRI in the gut with a significantly reduced number of leukocytes in animals deficient of γδ T cells compared to WT animals (Fig. 1A). As γδ T cells represent the major lymphocyte population among the IEL, overall lymphocyte numbers in the gut were slightly but statistically significantly reduced in animals deficient of γδ T cells compared to WT (Fig. 1B). Conceivably, *Tcrd*^*tm1Mom*^ mice displayed a significant decrease in their overall T cell fraction as compared to WT animals due to their lack of γδ T cells, especially in the IEL fraction (Fig. 1C). IRI did not impact the fraction of αβ or γδ T cells in each compartment (Fig. 1D,E).

### Neutrophil influx is decreased γδ T cell deficient mice

**Figure 2:**
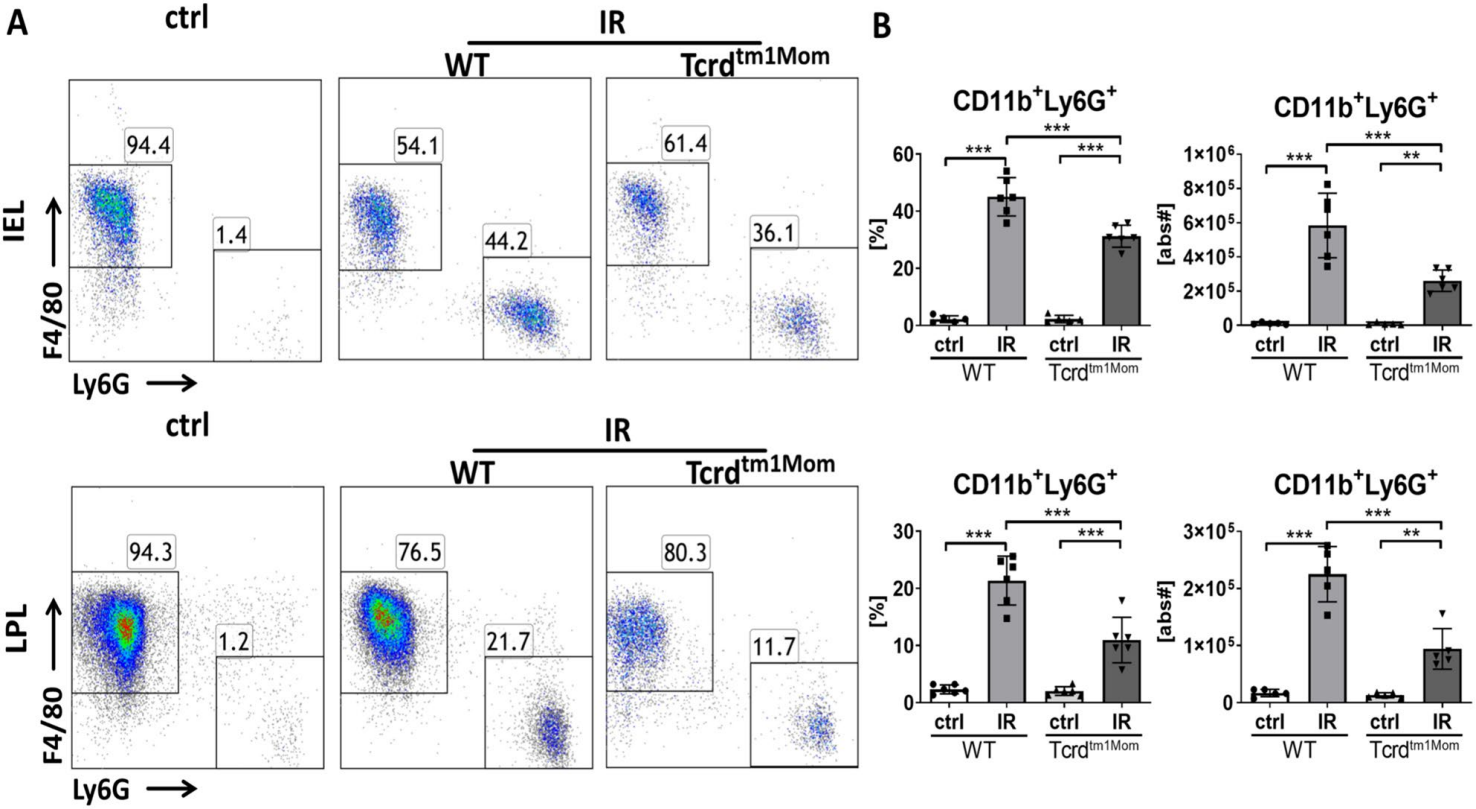
Neutrophil influx into the gut epithelium and lamina propria is reduced in γδ T cell deficient mice. Intraepithelial leukocytes (IEL) and lamina propria leukocytes (LPL) were isolated from small intestines and at least 1 × 10^5^ leukocytes per sample analyzed by flow cytometry in wild-type (WT) mice and mice deficient of γδ T cells (Tcrd^tm1Mom^) after intestinal ischemia reperfusion (IR) or sham operation (ctrl). **(A)** Flow cytometry of live CD45^+^CD11b^+^ leukocytes in IEL (upper row) and LPL (lower row) showed an increase of Ly6G^+^ neutrophils following IR, which is reduced in γδ T cell deficient mice compared to WT mice. **(B)** Percentage of CD11b^+^ myeloid cells among IEL (upper graph) and LPL (lower graph) in WT and Tcrd^tm1/mom^ following IR or ctrl treatment. Each data point represents the result of the analysis of an individual mouse. Bars represent group mean and error bar depicts ± SD. N = 6/group. *ns* not significant. ***p < 0.001. Data are representative of two independent experiments.

Neutrophils have been shown to be pivotal in mediating acute tissue damage in other organ models of IRI, while monocytes/macrophages extend the immediate injury^25,26^. Assessing the fraction of neutrophils and macrophages by flow cytometry revealed a massive influx of Ly6G^+^ neutrophils in the intestinal epithelium and, to a lesser extent, into the lamina propria (Fig. 2A). Statistical analysis of group data showed a diminished influx of neutrophils into the gut of γδ T cell deficient mice compared to WT animals (Fig. 2B).

### Diminished up-regulation of pro-inflammatory cytokines following IR in γδ T cell deficient mice

**Figure 3:**
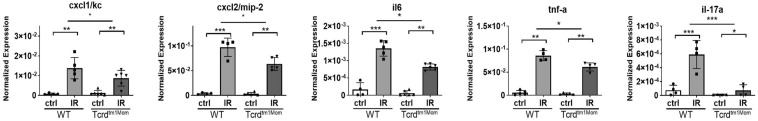
Decreased up-regulation of pro-inflammatory cytokines following IR in γδ T cell deficient mice. CXCL1/KC, CXCL2/MIP-2, IL-6, and TNF-α in the small intestine of wild-type (WT) mice and γδ T cell deficient mice of after intestinal ischemia reperfusion (IR) or sham operation (ctrl). The relative expression on mRNA level was assessed by quantitative reverse transcription PCR (RT-qPCR). relative Results are presented as mean expression value, normalized with GAPDH expression as a housekeeping gene. Data are presented as mean ± SD (n ≥ 4). *ns* not significant. ***p < 0.001. Data are representative of two independent experiments.

An inflammatory response detrimental to the affected organ is a key feature of IRI. In order to find possible effects due to a lack of γδ T cells in regard to the degree of inflammation, we assessed the expression of pro-inflammatory cytokines in the gut tissue by qPCR. Both chemokines CXCL1/KC and CXCL2/MIP-2 are major neutrophil attractants and mostly secreted by monocytes and macrophages. The archetypical pro-inflammatory cytokines IL-6 and TNF-α, are secreted by both T cells and macrophages, in order to regulate the inflammatory reaction in the acute phase response. Gene expression of CXCL1/KC, CXCL2/MIP-2, IL-6, TNF-α, as well asIL-17A, were all upregulated following IRI in the gut (Fig. 3). Compared to WT mice, γδ T cell deficient mice demonstrated a less pronounced up-regulation of these pro-inflammatory cytokines after IRI (Fig. 3).

### Ameliorated far distant organ injury in animals deficient of γδ T cells

**Figure 4:**
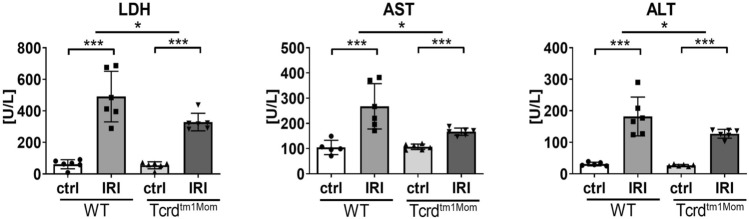
Weaker increase of serum LDH and transaminases following intestinal IRI in γδ T cell deficient mice. Levels of serum transaminases AST (left plot) and ALT (right plot) of wild-type (WT) mice and mice deficient of γδ T cells (Tcrd^tm1Mom^) after intestinal ischemia reperfusion (IR) or sham operation (ctrl). Each data point represents data of an individual mouse. Each bar represents group mean; error bar depicts SD. N = 5 6/group. *ns* not significant. ***p < 0.001. Data are representative of two independent experiments.

To test for a possible systemic effect after intestinal IRI, we measured ALT and AST levels in sera as markers of distant organ injury. Our results demonstrate a significant up-regulation of both enzymes following intestinal IR, which was less pronounced in γδ T cell deficient mice (Fig. 4).

## Discussion

Intestinal IRI is a significant clinical challenge with complex, multifactorial, and incompletely understood pathophysiology, but inflammation is known to be a common feature in different organ models. Recently, T cells gained interest as perpetuators of inflammation in a number of models of IRI^1–6^. Interestingly, studies in SCID mice, which lack all T and B cells, showed ameliorated intestinal IRI and thus presented convincing evidence that lymphocytes are involved in the pathogenesis. However, our previous study demonstrates that αβ T cells, the largest and conventional fraction carrying the T cell receptor, do not impact the acute phase of intestinal IRI and, therefore, may be considered as ‘innocent bystanders’^9^. Here, we extend these findings by demonstrating significant protection in γδ T cell-deficient mice compared with wild-type mice in the early phase of intestinal IRI.

Intestinal mucosal lymphocytes, which include IEL and LPL, are pivotal in the mucosal immune system, especially by providing immune surveillance of the epithelium^14^. Most of the IEL and LPL are T cells, which carry either the conventional αβ TCR or a γδ TCR. In all vertebrates, γδ T cells are the very first T cells to develop in the thymus but represent only a minor fraction of the circulating T cells in the body^10^. However, in epithelial tissue and especially in the gut, γδ T cells are present in much higher numbers, sometimes exceeding the number of conventional αβ T cells^14^. These highly conserved γδ T cells have been shown to play an important role in immune homeostasis of the gut, represent a first layer of defense, and protect from intestinal inflammation in a murine model of inflammatory bowel disease^27^.

Consistent with previous data from our^9^ and other groups^28^, we show that roughly half of the T cells among the IEL carry the γδ TCR and that the γδ T cell fraction is lower in the lamina propria (Fig. 1). Conceivably, mice lacking γδ T cells had significantly fewer absolute lymphocyte numbers in the gut epithelium compartment (Fig. 1). The same effect was seen also in the lamina propria, but did not reach significance (Fig. 1). Importantly, IRI did not impact lymphocyte number or distribution in the gut in WT or γδ T cell deficient mice following IRI in our model (Fig. 1), which confirms our previous data in a αβ T cell deficient intestinal IRI model^9^. These findings are in line with previous works demonstrating no or only minor differences regarding lymphocyte numbers in the gut after 1–3 h of intestinal IRI^7,29,30^. Therefore, the pronounced increase of leukocytes into the intestinal epithelium as well as into the lamina propria (Fig. 1) is due to an influx of myeloid cells with neutrophils representing the largest fraction (Fig. 2). Correspondingly, we observed an upregulation of CXCL1/KC, CXCL2/MIP-2, as major neutrophil attractants, as well as IL-6 and TNF-α, as archetypical pro-inflammatory cytokines in the gut after intestinal IRI (Fig. 3)^31^. We also observed a highly significant reduction of the expression of IL-17A following IR in mice lacking gd T cells (Fig. 3). Innate lymphoid cells have previously demonstrated to be the major producer of IL-17A in intestinal IRI and lack of IL-17 ameliorated intestinal IRI^32,33^. Our data conceivably supports this notion by demonstrating that the lack of the major producers of IL-17A also ameliorates intestinal IRI.

Strikingly, lack of γδ T cells resulted in a diminished leukocyte influx into the gut (Fig. 1), which we show to be caused by a reduced neutrophil influx (Fig. 2). In the corresponding assessment of cytokines, γδ T cell deficient mice demonstrated a less pronounced, but not completely abrogated, up-regulation of the assessed pro-inflammatory cytokines compared to WT mice (Fig. 3). We do not provide direct histological evidence of reduced tissue damage, but show reduced production of LDH as a marker indicating amelioration of intestinal IRI in mice lacking γδ T cells (Fig. 4). Besides γδ T cells, monocytes and macrophages are also capable of the secretion of neutrophil attractants, suggesting a contribution of those cell populations to the neutrophil influx in the post-ischemic gut^34^. We did not formerly address the question whether the lack of γδ T cells would influence cytokine production of other T cell fractions. In a previous report, however, we have demonstrated that lack of αβ T cells does not impact the cytokine profile in the early phases of intestinal IRI^9^.

Reperfusion of the ischemic intestine drives distant organ injury^35,36^, especially acute hepatocellular damage^9,37^. Thus we measured serum levels of transaminases to assess the systemic toxicity of the intestinal inflammatory reaction. Serum levels of transaminases were significantly upregulated after intestinal IR (Fig. 4). Interestingly, in mice lacking γδ T cells, the finding of ameliorated local gut inflammation following IRI was accompanied by significantly ameliorated levels of transaminases as compared to wild-type animals (Fig. 4). Confirmation of our results by functionally depleting WT mice of γδ TCR by mAb treatment would have been desirable in order to eliminate possible confounders as altered gut microbiota in genetically γδ T cell deficient mice. However, this is not feasible, as mAb treatment does not deplete but generates "invisible" gammadelta T cells^38^.

Taken together, the key findings of our study are that the genetic deficiency of γδ T cells in intestinal IRI reduces neutrophil influx, up-regulation of pro-inflammatory cytokines, and the release of transaminases as a marker of distant organ injury (Figs. 1, 2, 3, 4). These findings solve the conflicting results of earlier studies demonstrating amelioration of intestinal IRI in SCID mice, which lack all types of T and B cells^7,30,39^, and our previous study demonstrating that selective αβ T cells are functionally dispensable for initiating or perpetuating the initial inflammatory reaction^9^., In comparison to other immune cells, αβ T cells are relatively rare in the small intestine, whereas γδ T cells represent the majority of intraepithelial T cells. γδ T cells have pleiotropic effector functions, particularly at mucosal borders, including rapid innate-like immune responses^40,41^. Therefore, the isolated deficiency of γδ T cells in this study offers a feasible explanation for the beneficial effect on acute intestinal IRI. We conclude that in our acute model of intestinal IRI, T cells do, in fact, contribute to the damage, but it is important to differentiate the precise subset and function. αβ T cells, which have been unequivocally recognized as key mediators in the pathogenesis of IRI in other organs^1,9–11^, seem to represent innocent bystanders in intestinal IRI. In contrast, γδ T cells are considered to be more innate T cells, which was here supported by their rapid contribution to immune-mediated exacerbation of intestinal IRI. This is in line with recent studies in other models of IRI, demonstrating that γδ T cells make a significant contribution to IRI^17,42–44^.

In summary, we demonstrate that in the applied model of acute intestinal IR selective γδ T cell deficiency does significantly ameliorate the severity of the inflammatory response. We conclude that in this model of intestinal IRI, γδ T cells are critically involved in initiating and perpetuating the initial inflammatory reaction. However, further research is required to clarify the precise role of T cells in intestinal IRI and to better understand the complex interplay between these cells and the other immune cells in this model.
